# A Multi-Omics Study of Chicken Infected by Nephropathogenic Infectious Bronchitis Virus

**DOI:** 10.3390/v11111070

**Published:** 2019-11-16

**Authors:** Puzhi Xu, Ping Liu, Changming Zhou, Yan Shi, Qingpeng Wu, Yitian Yang, Guyue Li, Guoliang Hu, Xiaoquan Guo

**Affiliations:** 1 Jiangxi Provincial Key Laboratory for Animal Health, College of Animal Science and Technology, Jiangxi Agricultural University, Nanchang 330045, China; 18979191421@jxau.edu.cn (P.X.); Pingliujx@163.com (P.L.); zcm@jxau.edu.cn (C.Z.); taildog@163.com (Q.W.); yangyitian0810@163.com (Y.Y.); liguyue66@126.com (G.L.); 2 School of Computer and Information Engineering, Jiangxi Agricultural University, Nanchang 330045, China; shiyan0015@126.com

**Keywords:** metabolomics, transcriptomic, microbiomes, torrelation analysis, nephropathogenic infectious bronchitis virus

## Abstract

Chicken gout resulting from nephropathogenic infectious bronchitis virus (NIBV) has become a serious kidney disease problem in chicken worldwide with alterations of the metabolic phenotypes in multiple metabolic pathways. To investigate the mechanisms in chicken responding to NIBV infection, we examined the global transcriptomic and metabolomic profiles of the chicken’s kidney using RNA-seq and GC–TOF/MS, respectively. Furthermore, we analyzed the alterations in cecal microorganism composition in chickens using 16S rRNA-seq. Integrated analysis of these three phenotypic datasets further managed to create correlations between the altered kidney transcriptomes and metabolome, and between kidney metabolome and gut microbiome. We found that 2868 genes and 160 metabolites were deferentially expressed or accumulated in the kidney during NIBV infection processes. These genes and metabolites were linked to NIBV-infection related processes, including immune response, signal transduction, peroxisome, purine, and amino acid metabolism. In addition, the comprehensive correlations between the kidney metabolome and cecal microbial community showed contributions of gut microbiota in the progression of NIBV-infection. Taken together, our research comprehensively describes the host responses during NIBV infection and provides new clues for further dissection of specific gene functions, metabolite affections, and the role of gut microbiota during chicken gout.

## 1. Introduction

Gout is a urate crystal deposition disease that occurs when supersaturation of individual tissues with urate arises, leading to the construction of monosodium urate (MSU) crystals in and around the joints, abdomen, and organs [1,2]. It is worth noting that in addition to its occurrence in humans, gout is one of the common diseases that plague poultry and causes huge economic losses to the poultry industry worldwide [3]. Avian gout is commonly divided into visceral gout and joint gout, and the typical clinical pathology of visceral gout is hyperuricemia. According to reports, various aviaries from all over the world have visceral gout, which has become one of the most commonly diagnosed causes of fatality in poultry [4,5]. In the poultry industry, visceral gout can be caused by many factors, including vitamin A deficiency, high dietary calcium, renal insufficiency, chicken astrovirus (CAstV), and NIBV [4,6,7]. Currently, NIBV that causes visceral gout in chicken has become a research hotspot [6,8].

NIBV has become the most common IBV strain in the commercial poultry industry. It is a gamma Coronavirus in the Coronavirus family that exhibit strong renal tropism and has become one of the most familiar pathogens that induce visceral gout outbreaks [9,10,11]. Initially, some IBV strains have been called nephropathogenic IBV because the starting respiratory infection was followed by a heavy kidney infection. A large number of previous research findings indicate that clinical symptoms of nephropathogenic IBV strains infection include increased water consumption, depression, watery droppings, and significant mortality. Notably, necropsy of chicks that died during nephropathogenic IBV infection showed enlarged and pale kidneys and monosodium urate crystal deposition in the renal tubules and ureters [12]. According to histopathology, NIBV replicates on the epithelial surface of the kidney and causes particle degeneration, vacuolation, and desquamation of the tubular epithelium, as well as massive infiltration of heterophilic granulocytes in the stroma [13]. However, there is a substantial lack of understanding of the pathogenesis of NIBV infection. In addition, it is well known that the gut microbial community is closely associated with the progression of diseases, and Inoue et al. also confirmed that hepatitis C virus infection is associated with gut dysbiosis [14]. In our study, we focus on the cecum as organs of particular interest as they harbor the highest microbial cell densities (up to 10^11^ cells g^−1^) and are significant sites for recycling of urea, water regulation, and carbohydrate fermentations [15,16]. Therefore, we suspect that the gut microbiota may be associated with the progression of visceral gout induced by NIBV infection.

Nowadays, omics technology is increasingly being used to understand intricate biological systems and reveal the molecular characteristics behind complex cellular phenotypes [17,18]. Applications of omics platforms include the identification of genes (genomics), messenger RNA (mRNA, transcriptomics), proteins (proteomics), and metabolites (metabolomics). Moreover, the research of the gut microbiota (microbiomics) has brought increasing concern due to its important affection for different diseases [19]. Many scholars have used multi-omics analysis to make great progress in deciphering the pathogenesis of diseases, such as Alzheimer’s disease [20], familial type 1 diabetes [21], autoimmune diseases [22], and cardiovascular diseases [23]. However, to date, there has been very few omics analyses for the study of poultry diseases, and in particular, as far as we know, there is no omics analysis for the study of the pathogenesis of NIBV infection.

The antiviral and metabolic changes caused by viruses result in a highly complicated process that requires the coordination of diverse intertwined signalling and metabolic pathways. Therefore, a large-scale and comprehensive analysis of viral target tissues is needed to better understand the pathogenesis of the NIBV infection. We first replicated the visceral gout model by infecting chickens with NIBV and conducted an integrated transcriptomic, metabolomics, and microbiomics analyses. The characterization of these identified genes, metabolites, and microbiota clearly reflected the dynamic changes in the biological processes in chickens with NIBV infection. Overall, our research comprehensively describes the host responses during NIBV infection and provides new clues for further dissection of specific gene functions, metabolite affections, and the role of gut microbiota during chicken gout.

## 2. Materials and Methods

### 2.1. Virus Strains

The virulent IBV strain that was used was the SX9 strain which was isolated and stored by the College of Animal Science and Technology, Jiangxi Agriculture.

### 2.2. Experimental Design

**Figure 1:**
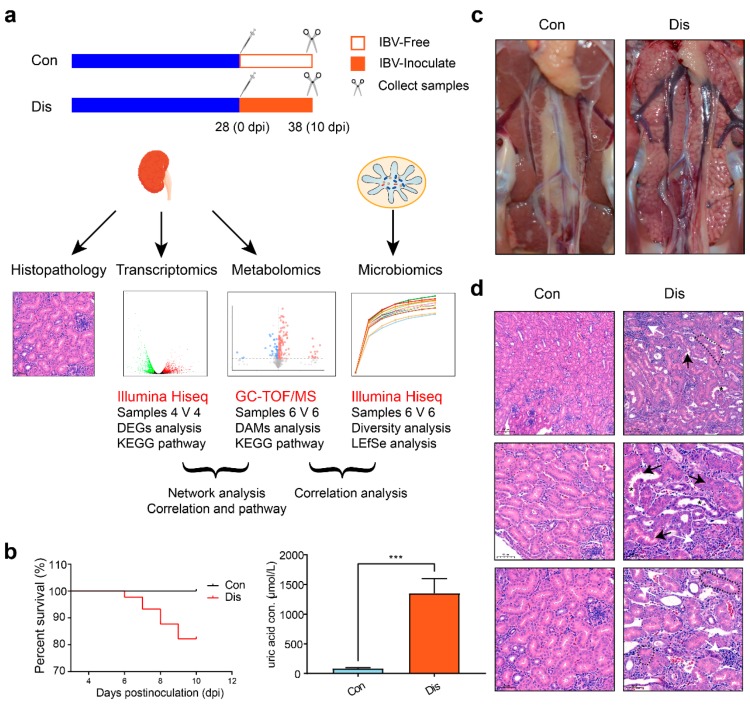
Changes in the kidney of chickens infected with nephropathogenic infectious bronchitis virus (NIBV). (**a**) Experimental design, including the analysis of transcriptomics, metabolomics, and microbiomics. (**b**) Analysis by Kaplan–Meier curve of 10 dpi survival rate in NIBV-infected chickens and uric acid concentrations in the serum. (**c**) Gross lesions in the kidneys. Kidney tissue of an uninfected control chicken (left). Obvious enlargement and urate deposition in the kidney of a chick infected with NIBV at 10 dpi (right). (**d**) Histopathological changes in the kidney of chickens infected with NIBV (H&E staining). The black arrow shows the shedding of kidney tubular epithelial cells and the white arrow shows the interstitial expansion and prominent inflammatory cell infiltration. The black asterisk shows the brush border that was lost in some segments of proximal tubules. The black delimited area shows the loss of the kidney tubular structure.

Two hundred forty Hy-Line Brown variety birds (1-day-old; Guohua Co. Ltd., Nanchang, Jiangxi, China) were stochastically split into two experimental rooms: A control room (Con, *n* = 120) and a diseased room (Dis, *n* = 120). Birds in each experimental room were randomly divided into three subgroups (30 birds for each subgroup), with ad libitum access to food and water. Each chicken of Dis groups was intranasally injected with 0.2 mL 10^5^ median embryo lethal doses of strain SX9 at 28 days of age [24], while the Con group intranasally received 0.2 mL of sterile physiological saline. At 38 days of age, four chicken randomly chosen per subgroups were euthanized by carbon dioxide inhalation, then dislocated their cervical vertebra. The samples in a group were pooled and dead birds were not used for analysis. Ten serum samples were randomly collected from surviving chickens in the Con and Dis groups before euthanasia that were used for uric acid test. Six biological replicates of kidney samples were extracted from each group making a total of 12 samples that were used for GC–TOF/MS analysis. Four biological replicates of kidney samples were collected from each group giving a total of eight samples that were used for RNA-seq analysis. Six biological replicates of cecal contents from each group were collected giving a total of 12 samples that were used for 16S rRNA gene sequencing analysis (Figure 1a shows the experimental design).

### 2.3. Histopathology

The isolated kidney tissues were fixed by immersion in 10% neutral formalin at room temperature for over 48 h. Tissues were then routinely processed; H&E staining was performed and a section per chicken was observed under the optical microscope.

### 2.4. Metabolomics Analysis

Metabolite extraction, metabolite derivatization, metabolite detection, and data analysis followed those of Yang et al. [25]. First, methanol (V_methanol_:V_chlorofrom_ = 3:1) was used as an extraction liquid, and L-2-chlorophenylalanine (1 mg/mL stock in dH_2_O) was added as an internal standard. The metabolites are then derivatized with the methoxy amination hydrochloride (20 mg/mL in pyridine) and the STFA regent (1% TMCS, *v*/*v*). Finally, GC–TOF/MS analysis was performed using an Agilent 7890 gas chromatograph system coupled with a Pegasus HT time-of-flight mass spectrometer. The energy was –70 eV in electron impact mode. After 6.04 min of solvent delay, mass spectrometry data were acquired in full-scan mode with an m/z range of 50–500 at a rate of 20 spectra per second.

Chroma TOF4.3X software (LECO Corporation, St. Joseph, MI, USA) and the LECO-Fiehn Rtx5 database were used for raw peak exacting, data baseline filtering and calibration, peak alignment, deconvolution analysis, peak identification, and integration of the peak area. SIMCA14 software package (Umetrics, Umea, Sweden) was used for further data analysis, including principal component analysis (PCA) and orthogonal projections to latent structures-discriminate analysis (OPLS-DA). In addition, commercial databases including Kyoto Encyclopedia of Genes and Genomes (KEGG, http://www.genome.jp/kegg/) and National Institute of Standards and Technology (NIST, http://www.nist.gov/index.html) were utilized to search for metabolic pathways. MetaboAnalyst (http://www.metaboanalyst.ca) was used for pathway enrichment analysis.

### 2.5. Transcriptomics Analysis

RNA preparation, library preparation, RNA-sequencing, and data analysis followed those of Yang et al. [26]. First, total RNA was extracted from each kidney sample individually using TRIzol reagent (Invitrogen, Burlington, ON, Canada). We further used the NanoDrop 2000 (Thermo, Waltham, MA, USA) to measure the RNA concentration, and the Agilent Bioanalyzer 2100 system (Agilent Technologies, CA, USA) to assess the RNA integrity. Library preparation was performed as described by Li et al. [27]. Briefly, a total amount of 1 μg RNA per sample was used for the RNA sample preparations. Sequencing libraries were generated using NEBNext UltraTM RNA Library Prep Kit for Illumina (NEB, Ipswich, MA, USA) and index codes were added to attribute sequences to each sample. The library fragments were purified with AMPure XP system (Beckman Coulter, Beverly, CA, USA). At last, PCR products were purified (AMPure XP system) and library quality was assessed on the Agilent Bioanalyzer 2100 system. Finally, the constructed cDNA libraries were sequenced by an Illumina Hiseq Xten platform.

Clean data of high quality were obtained from the raw data through in-house scripts by removing those containing adapters or poly-N sequences. All clean reads were aligned to the reference genome (https://www.ncbi.nlm.nih.gov/assembly/GCF_000002315.4/) using TopHat2 [28]. Cufflinks was used to calculate and analyze the gene expression levels, and FPKM (fragments per kilobase of exon per million fragments mapped) values of each gene were calculated based on the length of the gene and the fragments count mapped to this gene. Differential expression genes (DEGs) analysis was performed by using the DESeq R package (1.10.1). Only those genes with a FC (fold change) ≥ 2 and FDR (false discovery rate) < 0.01 were defined as DEGs. Furthermore, gene ontology (GO) enrichment analysis of DEGs was implemented by the GOseq R packages [29] and the enrichment analysis of DEGs in KEGG pathways was performed using KOBAS software [30]. In addition, the target seven DEGs in response to NIBV infection were chosen for validation using real-time quantitative PCR (RT-qPCR). The primer pairs for the selected genes were designed using Primer 5 and are shown in Table S1.

### 2.6. Microbiomics Analysis

Total genome DNA from samples was extracted from the cecal contents using CTAB/SDS method. DNA concentration and quality were determined and diluted to 1 ng/μL using sterile water. The 16S rRNA genes of each sample V3–V4 region were amplified. The primer set corresponding to primers 341F-806R with the unique barcode was applied for amplification (forward 341F: CCTAYGGGRBGCASCAG and reverse 806R: GGACTACNNGGGTATCTAAT). All PCR reactions were carried out with Phusion^®^ High-Fidelity PCR Master Mix (New England Biolabs, USA). The PCR products were detected on 2% agarose gel electrophoresis and bands of the desired size (approximately 400–450 bp) were chosen for further experiments. Sequencing libraries were generated using TrueSeq DNA PCR-free sample preparation kit (Illumina, San Diego, CA, USA) and index codes were added. Next, sequencing was performed on an Illumina HiSeq 2500 platform (Novogene Company, Beijing, China).

Based on their unique barcode, the paired-end reads were granted to samples. Those files were demultiplexed and quality filtered with quantitative insights into microbial ecology (QIIME) software (version 1.7.0) [31]. The chimera sequences were removed by using UCHIME algorithm and then the effective tags finally obtained [32]. Sequences were clustered to the same OTUs at 97% similarity by Uparse software (Uparse v7.0.1001). Moreover, the Greengene database was used to annotate taxonomic information and the multiple sequence alignment was conducted using the MUSCLE software (Version 3.8.31). Further, OTUs abundance information was normalized and subsequent analysis were all performed based on this output normalized data including alpha diversity, beta diversity, and linear discriminant analysis (LDA) effect size (LEfSe) analysis [33].

Heatmap for metabolomics, transcriptomics, and microbiomics data was generated using the pheatmap package. Corrplot package in R was used to visualize the correlations coefficient (Spearman method). Availability of data and materials: RNA-seq raw data are accessible through NCBI’ database: BioProject: PRJNA510170; 16S rRNA gene sequencing raw data are accessible through NCBI’ database: BioProject: PRJNA510025.

### 2.7. Ethics Approval and Consent to Participate

The institutional animal care and use committee of Jiangxi Agricultural University approved these animal experiments and all animal experiments adhered rigorously to the animal care guidelines of Jiangxi Agricultural University (approval ID: JXAULL-2017003; approval date: 8 March 2017). All the birds were sacrificed using carbon dioxide euthanasia, and all attempts were carried out to minimize the suffering of the animals.

## 3. Results

### 3.1. Clinical Signs and Pathology

The chicks inoculated with strain SX9 showed obvious clinical signs from 3 to 9 “day post-infection” (dpi). The NIBV-infected chickens were listless, huddled together, and displayed ruffled feathers, while no clinical symptoms similar to the above were observed in the Con group. The 10 dpi survival rate of all NIBV-infected chickens under analysis was 80%, and the uric acid level in the serum of the Dis group was much higher than that of the Con group (~1352 vs. ~85 μmol/mL, *n* = 10, *p* < 0.001, Student’s t-test, Figure 1b). We observed that kidney lesions were present in all Dis group chickens infected with SX9. At 10 dpi (mortality peak), the kidney parenchyma of the dead birds were pale, swollen, and mottled (Figure 1c). Histological examination revealed remarkable injuries in the kidney, including tubular epithelial cell detachment, loss of the kidney tubular structure, as well as interstitial expansion and prominent inflammatory cell infiltration (Figure 1d). These results indicate that the SX9 strain has strong renal tissue tropism and successfully replicates the chicken visceral gout model.

### 3.2. NIBV Infection Altered Metabolic Profiling in the Kidney of Chickens

**Figure 2:**
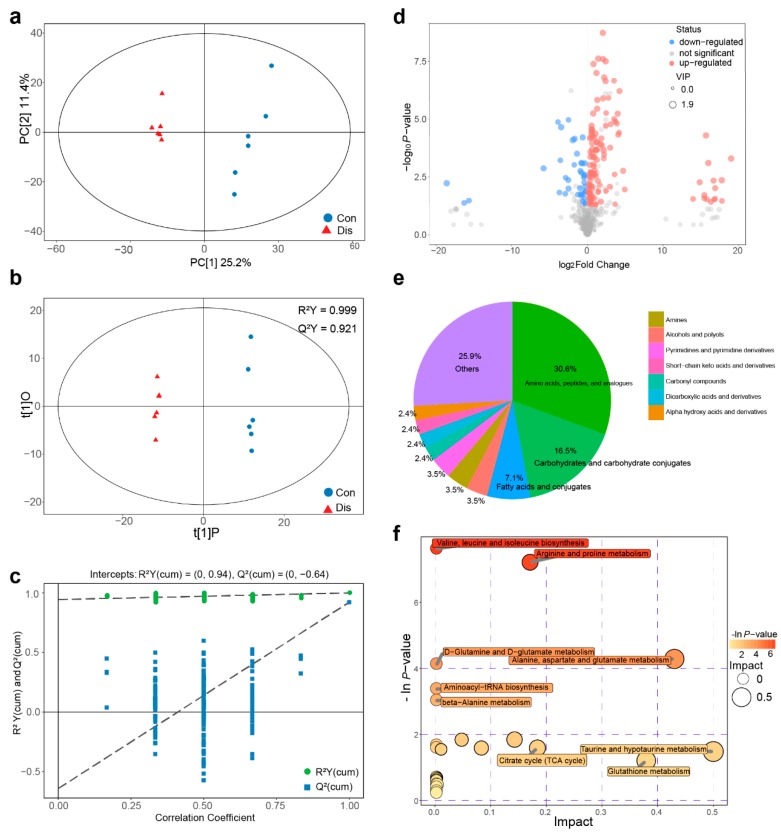
NIBV infection altered metabolic profiling in chicken kidneys. (**a**) Metabolic profile of the Con and Dis groups visualized by principal component analysis (PCA). The points represent the scores of biological replicates. (**b**) Metabolic profiles of the Con and Dis groups visualized by orthogonal projections to latent structures–discriminate analysis. The abscissa t [1] P represents the predicted principal component score of the first principal component, the ordinate t [1] O represents the orthogonal principal component score, and the scatter shape and colour represent different experimental groups. (**c**) Permutation test of orthogonal projections to latent structures-discriminate analysis (OPLS-DA) model for groups Dis vs. Con. (**d**) Volcano plot of differential metabolites, each point represents a metabolite and the point size represents the VIP value. (**e**) The category of the differential metabolites. (**f**) Pathway analysis for groups Dis vs. Con; each bubble in the bubble diagram represents a metabolic pathway.

To explore the metabolic pathway alterations associated with NIBV infection, we used a GC–TOF/MS-based metabolomics method to examine metabolite alterations in the kidney. A total of 519 valid peaks were identified in the total ion current profiles. To compare the metabolite composition of the Con and Dis groups, PCA models were tested (Figure 2a). OPLS-DA was conducted to determine whether NIBV infection influenced the metabolic pattern (Figure 2b) and a 7-fold cross validation was further applied to estimate the robustness and predictive ability to validate the model (Figure 2c). The results of PCA and OPLA-DA analysis showed that there was an obvious separation between the content of the Con and Dis groups, revealing significant changes in the concentrations of metabolites in the kidney induced by NIBV infection. All samples fell within the 95% (Hotelling’s T-squared ellipse) confidence interval.

The differential metabolites between Dis and Con groups were the key to explaining the occurrence of gout in chickens under NIBV infection. A total of 160 metabolites displayed significantly different levels based on VIP > 1 (variable importance for the projection, OPLS-DA model) and *p*-value < 0.05 (Student’s t-test). The lists of differential metabolites are shown in Table S2 and its volcano plot are shown in Figure 2d. There were 90 annotated metabolites in all 160 differential metabolites, and its categories are shown in Figure 2e. As Figure 2e shows, the differential metabolites were mainly classified as amino acids, carbohydrates, fatty acids, and conjugates. Among those annotated differential metabolite profiles which were displayed in heat maps (Figure S1), 65 metabolites were significantly upregulated and 25 were significantly downregulated in the Dis group compared to the Con group.

In addition, the pathway enrichment results are shown in Figure 2f. The analysis revealed that the differential metabolites participated in six target pathways (*p* < 0.05), including valine, leucine and isoleucine biosynthesis, arginine and proline metabolism, alanine, aspartate and glutamate metabolism, D-glutamine and D-glutamate metabolism, aminoacyl-tRNA biosynthesis, and beta-alanine metabolism. Among them, there are two pathways with an impact factor > 0, including valine, leucine and isoleucine biosynthesis (impact value = 0.43), and arginine and proline metabolism (impact value = 0.17). These pathways are related to amino acid metabolism and glycometabolism, which are involved in protein synthesis and energy production, respectively. Moreover, metabolomics highlighted N-formyl-L-methionine, a well-known agent for kidney injury for its effects on the increase of the vascular tone/resistance and reduction of renal perfusion [34], as the most relevant metabolite alteration in NIBV-infected chickens (VIP = 1.894). Undoubtedly, the uric acid level in the Dis group was significantly upregulated compared with the Con group (VIP = 1.798, 4.62-fold).

### 3.3. NIBV Infection Altered Transcription Profiling in the Kidney of Chickens

To study the gene expression alterations of chicken’s kidney under NIBV infection, cDNA libraries from two groups were subjected to Illumina sequencing. A total of 81.18 Gb clean data was obtained, and the Q30 base percentage of each sample was not less than 90.37%. From the mapping results, the mapping efficiency between the reads and the reference genome of each sample was between 75.59% and 79.29%. The detail of the sequencing and mapping results is provided in Table S3.

**Figure 3:**
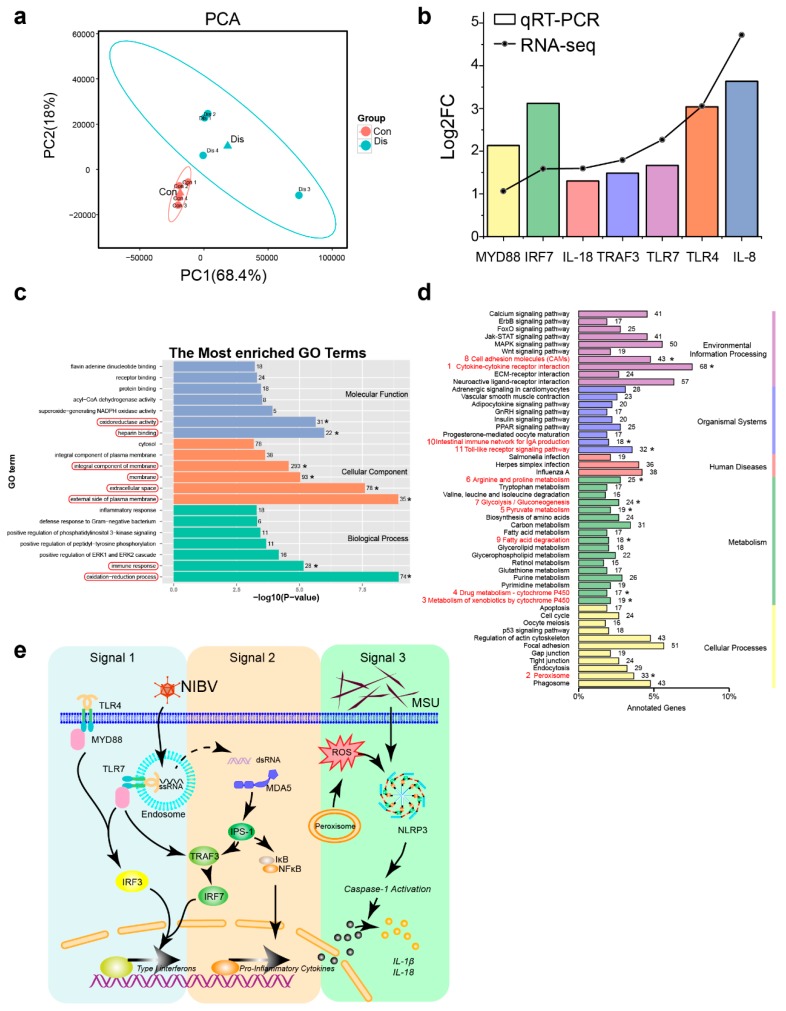
Transcriptome analysis of chickens with NIBV infection. (**a**) PCA plot showing the distinction between Dis and Con group based on gene expression. The blue and red triangles represent the mean coordinates (center point) of the Dis and Con group, respectively. (**b**) RT-qPCR validation of differentially expressed genes. (**c**) Gene ontology (GO) enrichment analysis of Differential expression genes (DEGs) between Con group and Dis group shows the 20 most enriched GO terms * represents the significantly changed GO terms with *p*_adj_ < 0.05 and the red bar is for highlighting. (**d**) Kyoto Encyclopedia of Genes and Genomes (KEGG) pathway classification enrichment analysis of DEGs. The y-axis and x-axis indicate the KEGG pathway and the percentage of annotated genes, respectively. The 11 significantly enriched pathways are listed with red serial number. (**e**) The schematic diagram representing important processes in chickens affected by NIBV infection, which activate innate immunity to antiviral infection. (Signal 1) Toll-like receptor (TLR) signalling is activated by the nucleic acid. TLR7 which is located in endosomal membrane induces production of type I interferon and proinflammatory cytokines via MyD88-NF-κB/IRF signalling pathway. (Signal 2) RIG-I-like receptor signaling is activated by dsRNA intermediates produced during NIBV replication. MDA5 recognizes a complementary set of cytosolic viral dsRNA ligands which can also activate NF-κB signalling. (Signal 3) NOD-like receptor (NLRs) signalling is activated by monosodium urate (MSU) and reactive oxygen species (ROS). The activation of TLRs and the ROS accumulation activates the NLRP3 inflammasome, resulting in proteolytic cleavage of caspase-1 and the maturation of IL-18 and IL-1β, and MSU has been identified to activate inflammasome complex.

PCA analysis of RNA-seq replicates from the kidneys of Dis and Con group revealed a great deal of variability (Figure 3a). A total of 2868 genes were differentially expressed in the chicken’s kidney with NIBV infection compared to Con group, in which 1521 genes were upregulated and 1347 genes were downregulated. Then, we screened seven DEGs for RT-qPCR analysis to validate the accuracy of the RNA-seq data, and the result (Figure 3b) showed that the general trends of the selected genes were consistent which proved the reliability of our RNA sequencing profiling.

GO (http://www.geneontology.org) project was applied to explore the potential biological functions of DEGs. In this study, the 20 most enriched GO terms classified by molecular function (MF), cellular components (CC), and biological processes (BP) terms were listed in Figure 3c. Among them, heparin binding, oxidoreductase activity, external side of plasma membrane, extracellular space, membrane, integral component of membrane, oxidation-reduction process, and immune response were the significantly enriched GO terms (*p*_adj_ < 0.05) in chickens with NIBV infection. To further study the biochemical metabolic pathway and signal transduction pathways related to NIBV infection, KEGG analysis and pathway enrichment analysis were conducted in the KEGG pathway database. There were 901 of the 2868 DEGs that were annotated to 177 pathways in KEGG analysis. The level-2 KEGG classes are shown in Figure 3d, and the 11 significant enriched pathways (Corrected *p*-value < 0.05 as determined by a Fisher’s exact test) are marked with red number. In environmental information processing pathways which include cytokine–cytokine receptor interaction and cell adhesion molecules (CAMs) were dramatically regulated. In cellular processes pathway, peroxisome was dramatically affected. In metabolism pathways, metabolism of xenobiotics by cytochrome P450, drug metabolism-cytochrome P450, pyruvate metabolism, arginine and proline metabolism, glycolysis/gluconeogenesis, and fatty acid degradation were significantly regulated. In organismal systems pathways, intestinal immune network for IgA production and Toll-like receptor signaling pathway were significantly regulated. Furthermore, most of these pathways of the upregulated genes enriched were immune system-related pathways, and “cytokine-cytokine receptor interaction” was the most represented pathway. Among these pathways we found upregulated, “Toll-like receptor signalling pathway”, “NOD-like receptor signalling pathway” and “RIG-I-like receptor signalling pathway” are pattern recognition signal receptor pathways involved in innate immunity. Simultaneously, the transcriptome showed that the “peroxisome” and amino acid metabolite pathways were suppressed in chickens infected with NIBV. Based on the above results, we predicted a schematic diagram of important pathways for chickens in the NIBV infection processes (Figure 3e).

### 3.4. NIBV Infection Resulted in Gut Microbiota Dysbiosis

**Figure 4:**
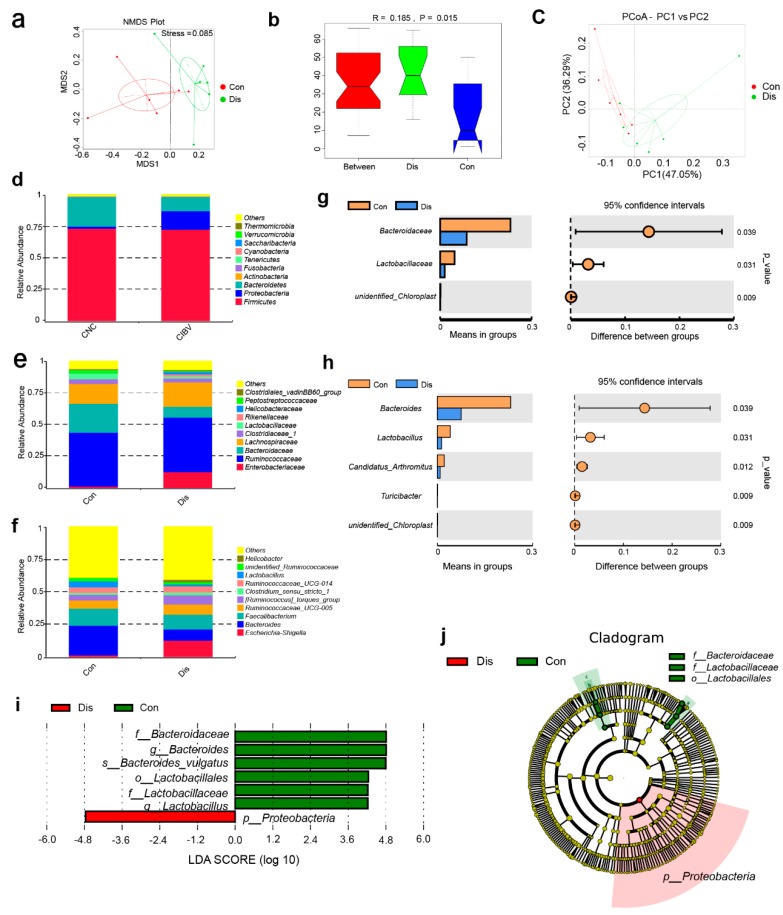
NIBV infection resulted in gut microbiota dysbiosis. (**a**) Nonmetric multidimensional scaling (NMDS) showing the difference in bacterial communities according to Bray–Curtis distance. (**b**) Analysis of similarity (ANOSIM) analysis of the beta diversity of the samples significantly separating the groups when R > 0 and *p* < 0.05. (**c**) Principle coordination analysis (PCoA) plot of similarities between the different groups. The top 10 relative abundances of bacteria at the phylum (**d**), family (**e**), and genus (**f**) levels in cecal content samples from the Con and Dis groups. The difference in species analysis between the Con and Dis groups at the family (**g**) and genus (**h**) levels according to a t-test. (**i**) The linear discriminant analysis (LDA) value distribution histogram shows species with an LDA ≥ 4. (**j**) Cladogram showing the most differentially abundant taxa identified by linear discriminant analysis effect size (LEfSe).

Considering that the intestinal tract is an important organ for lowering serum uric acid concentrations, 16S rRNA sequencing was performed and demonstrated marked alterations of the gut microbial communities in the Dis group. The rarefaction analysis curves for each group were near saturation, revealing that the sequencing data had a great quality and that certainly few new species were present (Figure S2). NMDS (nonmetric multidimensional scaling, Figure 4a), ANOSIM (analysis of similarity, Figure 4b), and PCA analysis (Figure 4c) confirmed the significant separation of the groups, indicating clear differences in microbial composition in the Con and Dis groups.

The top 10 microbes at the phylum (Figure 4d), family (Figure 4e), and genus (Figure 4f) levels are shown and indicate significant variations of the microbial community. Analyses of the microbiota composition at the phylum level showed a dominance of *Firmicutes*, *Proteobacteria,* and *Bacteroidetes* in both groups. We observed a decrease in the family *Bacteroidaceae* (*p* = 0.039, t-test), family *Lactobacillaceae* (*p* = 0.031, *t*-test) and the genus *Bacteroides* (*p* = 0.039, t-test), genus *Lactobacillus* (*p* = 0.031, t-test), genus *Candidatus Arthromitus* (*p* = 0.012, *t*-test), and genus *Turicibacter* (*p* = 0.009, *t*-test) in the Dis group (Figure 4g,h). LEfSe was performed to identify significant microbiota composition differences between the groups. Seven differentially represented core major groups were identified (Figure 4i,j). Differentially abundant phylum detected showed that *Proteobacteria* (LDA = 4.7644853231) phylum was most dominantly present in Dis group. At the genus level, the microbiota of the Con group was enriched with *Bacteroides* (LDA = 4.80848153017) from the *Bacteroidetes* phylum and *Lactobacillus* (LDA = 4.22406025945) from the *Firmicutes* phylum. At the species level, the microbiota of the Con group was enriched with *Bacteroides vulgatus* (LDA = 4.79658975077) from the *Bacteroidetes* phylum.

### 3.5. Correlation Analysis of Metabolomics and Transcriptomics Data

**Figure 5:**
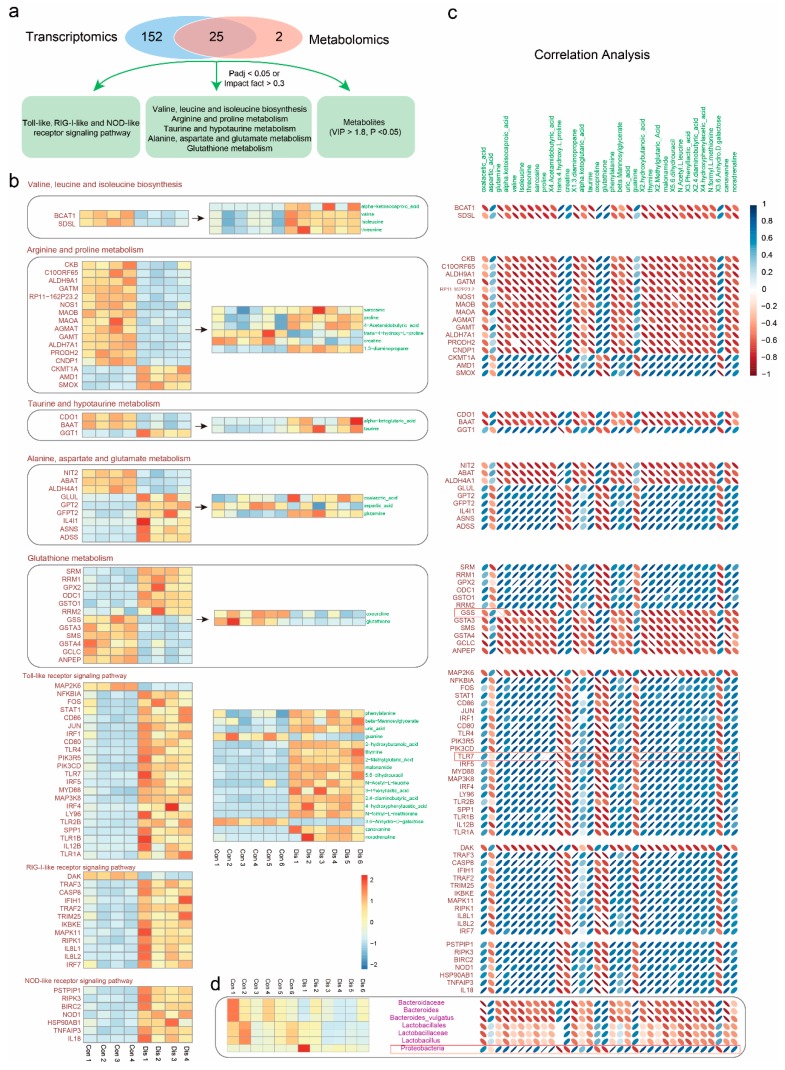
The significant correlation between DEGs, differential metabolites, and discriminative features of gut microbiota. (**a**) Overlap enriched pathways between metabolomics and transcriptomics. (**b**) Heat map of DEGs and metabolites used for correlation analysis. Heat map of DEDs and differential metabolites involved in overlap pathway were shown in the grey box, respectively. (**c**) Spearman’s rank correlation analysis. The color indicating the sign of the correlation, and the shape indicating the strength (narrower ellipses = higher correlations). The red bar is for highlighting. (**d**) Heat map of the discriminative features of gut microbiota and correlation plot of spearman correlation analysis between differential metabolites and discriminative features of gut microbiota.

The above data describe that NIBV infection alters kidney gene expression and metabolic pathways (Figure 5a). We filtered out five overlapping pathways in the transcriptome and metabolome pathway enrichment analysis, including valine, leucine and isoleucine biosynthesis, arginine and proline metabolism, taurine and hypotaurine metabolism, alanine, aspartate and glutamate metabolism, and glutathione metabolism. In addition, the DEGs in significantly enriched pathways associated with innate immunity and differential metabolites with VIP > 1.8 were also chosen for correlation analysis (Tables S4 and S5, respectively). Differences in those pathways—associated with DEGs and differential metabolites were shown with heatmap in Figure 5b. Then, we analyzed the Spearman correlation between genes and metabolites in those pathways, and the result is shown in Figure 5c. As shown in Figure 5c, there was a positive correlation between *Tlr7* (TLR, Toll-like receptor) and proline (R = 1, *p* = 0), isoleucine (R = 0.9524, *p* = 0.0098), threonine (R = 0.9762, *p* = 0.0035), and valine (R = 0.9762, *p* = 0.0035). Conversely, strong negative correlations were found between glutathione synthetase (GSS) and glutamine (R = −1, *p* = 0). Interestingly, we observed that most genes related to innate immune responses had strong positive correlations with differentially abundant metabolites, such as amino acids and fatty acids.

### 3.6. Correlation Analysis of Metabolomics and Microbiomics Data

The gut microbiota is deliberated a massive “organ” able to perform complex functions and thereby produce a myriad of differentially abundant metabolites. To investigate the functional correlation between the gut microbiome changes and host metabolome alterations, a correlation plot was visualized by calculating Spearman’s correlation coefficients (Figure 5d). The result indicated that clear correlations could be identified between altered metabolic profiles and modulated gut microbiomes (Table S6). Of particular note, some metabolites, including trans-4-hydroxy-L-proline, guanine, and 3,6-anhydro-D-galactose, which decreased in the kidney of NIBV-infected chickens, were negatively correlated with the presence of the phylum *Proteobacteria*. Furthermore, other metabolites, including canavanine, 2,4-diaminobutyric acid, 5,6-dihydrouracil, malonamide, thymine, phenylalanine, 1,3-diaminopropane, 4-acetamidobutyric acid, proline, threonine, isoleucine, valine, and oxalacetic acid, which increased in the kidney of NIBV-infected chickens, were positively correlated with the presence of the phylum *Proteobacteria*. These observations indicated that the significantly modulated gut microbiota was correlated with host metabolic disorders.

## 4. Discussion

In our study, the three pathways included in the activation of the innate immune system, the Toll-like receptor signalling, RIG-I-like receptor signalling, and NOD-like receptor signalling pathways, that are the most important three parts of the pattern-recognition receptor (PRR) signalling pathway are usually activated in response to infections to stimulate inflammatory responses [35,36,37]. In the Toll-like receptor signalling pathway (Figure 3f, Signal 1), a series of upregulated genes were noticed, including TLR4, TLR7, MyD88, IRF5, TRAF3, and IRF7. It was already reported that TLR7 primarily recognizes single-stranded RNA (ssRNA) sequences of RNA viruses that enter endosomes by endocytosis [38,39,40]. NIBV used in this experiment is a single-stranded positive sense RNA virus. Thus, the expansion of TLR7 is pivotal for the recognition of NIBV and variable functions of the Toll-like receptor signalling pathway. Moreover, a number of viral glycoproteins have been shown to act as viral PAMPs (Pathogen-associated molecular pattern) that bind to and activate TLR4, leading to IFN-β and/or proinflammatory cytokine expression (such as SARS coronavirus) [41]. Ru Liu-Bryan’s study has established that host expression of TLR2, TLR4, and their intracellular adapter protein MyD88 is a major mediator of MSU crystal-induced inflammation [42]. This explains the reason for the increase in *Tlr*4 transcription levels in this experiment. In addition, the transcriptomic analysis showed that NIBV infection also activated the RIG-I-like receptor signalling pathway (Figure 3f, signal 2), which included the transcriptional upregulation of genes such as MDA5, IPS-1, TRAF3, and IκB. This induction may be due to MDA5 acting as a double-stranded RNA (dsRNA) sensor to trigger an innate immune response against viral infection [43,44,45], while coronaviruses can produce dsRNA intermediates during replication [46]. RLRs can not only be expressed in cells infected with various viruses but also directly recognize and perceive virus products and virus particles present in the cytosol outside of the endosomes [47]. Therefore, we suspect that the RIG-I-like receptor signalling pathway has a greater role than Toll-like receptor signalling in recognizing NIBV infection. The activation of the Toll-like receptor signalling and RIG-I-like receptor signalling pathways results in the production of chemokines and other cytokines that induce a proinflammatory response and tissue destruction. In our study, several features that are familiar to chickens infected with NIBV are consistent with the immunopathological disease. These features include the pathological damage of kidney and the presence of increased transcription of chemokines and other cytokines, such as IL-8 (interleukin 8), IL-18 (interleukin 18), and TGF-β (transforming growth factor β).

Peroxisomes act to eliminate a microbial infection by modulating the canonical innate immunity pathways through ROS signalling [48]. Several enzymes with antioxidant activity in the peroxisome are involved in neutralizing ROS to protect the cells from ROS damage. Among these enzymes, catalase (CAT), superoxide dismutase 1 (SOD1), peroxiredoxin 5 (PRDX5), glutathione S-transferase kappa 1 (GSTK1), dehydrogenase/reductase member 4 (DHRS4), and epoxide hydrolase 2 (EPHX2) are included [49,50]. Multiple viruses have been shown to use different mechanisms to reduce peroxisome numbers or interfere with their functions. In our study, the “peroxisome” was the most important pathway according to the downregulated gene enrichment analysis, which includes CAT, SOD1, DHRS4, and EPHX2 that belong to the peroxisome antioxidant defence system, while the catalase activity was decreased both in kidney and serum (Figure S3). Thus, decreased abundance and/or impaired function of the peroxisome could potentially cause endogenous elevation of ROS. ROS may either straightly trigger NLRP3 inflammasome assemblage or be indirectly sensed through cytoplasmic proteins that modulate inflammasome activity (Figure 3f, signal 3). The NLRP3 inflammasome senses pathogens or danger signals to promote the maturation of cytokines such as IL-18 [51]. The release of active IL-18 engages IL-18 receptor-harbouring cells and promotes inflammatory responses [52]. In addition, uric acid has been reported as another well-established activator of NLRP3 that is usually generated via xanthine oxidase (XOD), accompanying the generation of O_2_^•−^ [53,54]. Consistently, in this study, we detected a significant increase in XOD activity in serum (Figure S3) and a significant increase in serum uric acid levels (Figure 1b). These results indicate that NIBV infection can activate the inflammatory response by inducing severe ROS accumulation.

The kidney is responsible for the elimination of 70% of the daily UA production [55]. ATP-binding cassette transporter, sub-family G, member 2 (ABCG2) is a high-capacity urate exporter that is located in the brush border membrane of kidney proximal tubule cells (S3 segment). ABCG2 dysfunction results in extra-renal urate under-excretion and is a common mechanism of hyperuricemia [56,57,58]. In the present study, the ABCG2 mRNA was downregulated in the model group chicken kidneys, partially explaining the significantly increased uric acid levels caused by NIBV infection. In addition to being caused by insufficient urate excretion, hyperuricemia can also be caused by excessive production of uric acid [59]. We found a high level of glutamine, which enter the purine metabolic pathway as a raw material for uric acid synthesis. The high level of glutamine can be explained by two reasons. First, the lack of mRNA abundance of the gene GSS in the kidney tissue of the model group inhibited the conversion of glutamine to glutathione. A significant negative correlation between GSS and glutamine has confirmed this finding. Second, the transcription level of the gene glutamic pyruvate transaminase 2 (GPT2) in the model group chickens’ kidneys increased significantly. GPT2 is a pyridoxal enzyme that promotes the conversion of α-ketoglutarate to glutamate [60]. Of note, α-ketoglutarate is an intermediate in the TCA cycle, and glutamate can be reversibly converted to glutamine by glutamine synthetase. Together, these data point to key genes and metabolites related to elevated uric acid synthesis, which provides us with new insights into host response to NIBV infection.

The present study highlights the correlation between differential expression of genes and differential abundance of metabolites in significantly enriched pathways, and the results showed that those differentially abundant metabolites that map in amino acid metabolism pathways have strongly positive correlations with DEGs related to innate immune responses. It is well known that organisms fuel or instruct the immune response to pathogen threats by modulating metabolic pathways [61]. Innate and acquired immune systems are regulated by a highly interactive network of chemical communications, which include the synthesis of the antigen-presenting machinery, immunoglobulins, and cytokines. This network is highly dependent on the sufficient availability of amino acids. Thus, amino acids affect immune responses either directly or indirectly through their metabolites [62]. In our study, the correlation analysis highlighted a significant positive correlation between *Tlr7* and proline, isoleucine, threonine, and valine. According to reports, isoleucine could maintain the development of immune organs and cells and stimulate the secretion of immune molecule substances in humans and animals [63,64], valine could improve dendritic cell function [65], and it has been proved in vitro that threonine plays a key role in lymphocyte proliferation and immunoglobulin production [66]. We observed an increase in the level of amino acids enriched in proline, leucine, and isoleucine biosynthesis pathway, including isoleucine, valine, and threonine. Therefore, changes in these amino acid metabolism pathways may occur through the activation of innate immunity to enhance the body’s antiviral response. Although there are many studies on amino acids and immunity, the modulation of amino acid metabolism in innate immune responses is still poorly known and deserves further study.

Previous research has confirmed that the dysfunction of the gut microbiome is tightly associated with kidney and liver diseases, including liver cirrhosis [67], liver cancer [68], and chronic kidney disease [69]. The gut microbiome incorporates a wide variety of commensal microbiota and has a large effect on the health of individuals. A community-wide effect involving the gain and loss of microbial populations and changes in general metabolic functions always occurs under pathological conditions. Our results showed that the abundance of *Bacteroides*, *Lactobacillus,* and *Bacteroides vulgatus* were significantly reduced in the cecal microbiota of NIBV-infected chickens based on LEfSe analysis. Similar to many other *Bacteroides* species, especially *Bacteroides vulgatus* which was shown to promote intestinal homeostasis, the *Bacteroides vulgatus*-mediated induction of semimature dendritic cells is associated with inflammation silencing [70,71]. Thus, decreased abundance of *Bacteroides vulgatus* promotes intestinal flora imbalance and activation of the immune system. Furthermore, it is well accepted that *Lactobacillus* may lower serum uric acid levels by reducing intestinal absorption of purines in humans [72]. Therefore, a decrease in the abundance of *Lactobacillus* in the caecum of the model group infected with NIBV further promoted an increase in uric acid levels in serum. The results of our correlation analysis further confirm this point, that is, the negative relationship between the abundance of *Bacteroides vulgatus*, *Lactobacillus* and the uric acid concentration. However, the mechanism of NIBV infection leading to a decrease in their abundance still needs further study. Taken together, multitudinous studies to date endorse the concept that a bloom of *Proteobacteria* in the gut reflects gut dysbiosis or an unstable gut microbial community [73]. In view of balanced gut microbiota with high stabilization that has symbiotic relationships with the immune system of the host, which is capable of suppressing the uncontrolled expansion of *Proteobacteria*, the increase in the abundance of *Proteobacteria* in this experiment may be closely related to the immune and metabolic changes caused by NIBV infection.

This report is the first time that multi-omics approach was employed to profile the metabolic changes and immune responses in kidney, as well as the effects on the intestinal microbiome during NIBV infection. Our results showed that NIBV significantly increased uric acid synthesis, inhibited the function of peroxisomes, and significantly elevated the pattern recognition receptor signalling pathways. In summary, our study comprehensively describes the host responses during NIBV infection and provides new clues for further dissection of specific gene functions, metabolite affections, and the role of gut microbiota during chicken gout.

## Supplementary Materials

The following are available online at https://www.mdpi.com/1999-4915/11/11/1070/s1. Figure S1: Heat map of annotated significant differential metabolites. Note: The increasing gradient of red color indicate higher upregulation, while an increasing gradient of blue color indicate higher downregulation. Figure S2: Rarefaction curves of the species number in the Con and Dis groups. The curve in each group is nearly smooth when the sequencing data set is sufficiently large (*n* = 6 per group). Figure S3: The catalase activity (a, b), xanthine oxidase activity (c, d) in kidney and serum. Table S1: Primers of selected genes for RT-qPCR. Table S2: Differentially abundant metabolites identified by GC–TOF/MS. Significant differences were declared at the level of *p*-value < 0.05 and VIP > 1. Table S3: Basic summary of sequence and sequencing reads mapping to reference genome. Table S4: DEGs in enriched pathways for correlation analysis. Table S5: Differentially abundant metabolites in enriched pathways for correlation analysis. Table S6: The relative abundance of seven discriminative features (LDA score ≥ 4) identified by LEfSe analyse.
