# Supplementary material for: A Multi-Omics Study of Chicken Infected by Nephropathogenic Infectious Bronchitis Virus
Source: Viruses. 2019 Nov 16;11(11):1070. doi: 10.3390/v11111070 (PMC6893681; doi:10.3390/v11111070)
Supplement: Supplementary file 1 [file viruses-11-01070-s001.zip › Supplementary files-for conversion/Table S1.pdf]

**Supplementary Table S1. Primers of selected genes for RT-qPCR.**

| Gene | Forward primer | Reverse primer |
|---|---|---|
| MYD88 | 5' TTGACTTCTGCATGGGTCCT 3' | 5' TTGCTCCACAGTCACCAGAT 3' |
| IRF7 | 5' AGTACTGGTACGAGCAGGTG 3' | 5' TCAGTCTGTCTGCATGTGGT 3' |
| IL-18 | 5' GTGTGTGCAGTACGGCTTAG 3' | 5' TCCACTGCCAGATTTCACCT 3' |
| TRAF3 | 5' CCAGCTCTCAGCAGCAGGAGACA 3' | 5' TCAGCACGAGGACACGGAAGC 3' |
| TLR7 | 5' CCAGATGCCTGCTATGATGC 3' | 5' TCAGCTGAATGCTCTGGGAA 3' |
| TLR4 | 5' CATACAAGCCACTCCAAGCC 3' | 5' AGGATTTCCAGGGCTGAGTC 3' |
| IL-8 | 5' GCAAGGTAGGACGCTGGTAA 3' | 5' GCGTCAGCTTCACATCTTGA 3' |
| GAPDH | 5' TGGCATCCAAGGAGTGAGC 3' | 5' GGGGAGACAGAAGGGAACAG 3' |
