# Supplementary material for: A Multi-Omics Study of Chicken Infected by Nephropathogenic Infectious Bronchitis Virus
Source: Viruses. 2019 Nov 16;11(11):1070. doi: 10.3390/v11111070 (PMC6893681; doi:10.3390/v11111070)
Supplement: Supplementary file 1 [file viruses-11-01070-s001.zip › Supplementary files-for conversion/Table S2.pdf]

Supplementary Table S2. Differentially abundant metabolites identified by GC-TOFMS. Significant differences were declared at the level of *P*-value < 0.05 and VIP > 1.

Peak	Similarity	rt	Count	Mass	MEAN Dis	MEAN Con	VIP	P-VALUE	Q-VALUE	FOLD CHANGE	LOG FOLDCHANGE
Linoleic acid methyl ester	602	20.0213,0	25	67	0.000555538	0.030774116	1.679388506	0.001338807	0.003872007	0.018052111	-5.791688621
3-phosphoglycerate	659	17.0399,0	17	357	0.000414487	0.004936229	1.198542882	0.00060567	0.00203372	0.08396843	-3.574009171
3,6-Anhydro-D-galactose 3	749	16.4613,0	21	231	0.007564958	0.083696122	1.878249401	2.22052E-05	0.000173177	0.090385999	-3.467756876
Biuret 3	246	12.3701,0	15	69	0.002071647	0.015663249	1.26324578	0.016897573	0.027064497	0.132261616	-2.918533665
Galactonic acid	780	18.9492,0	14	292	0.002891791	0.02122392	1.215498533	0.003424965	0.008039255	0.136251519	-2.875655778
1,2,4-Benzenetriol	368	14.7575,0	15	254	0.00261417	0.014769748	1.193008102	1.07566E-05	0.000111459	0.1769949	-2.498220302
glutathione 2	388	22.005,0	16	174	0.007186624	0.040509588	1.654233761	0.010087805	0.018906185	0.177405513	-2.494877252
O-phosphonothreonine 4	526	17.4674,0	19	356	0.02198677	0.070486669	1.668727136	7.06561E-05	0.000363453	0.311928063	-1.680714745
O-Phosphoserine 1	754	17.3578,0	27	356	0.071772511	0.185957671	1.591958847	0.000313071	0.001178763	0.385961548	-1.373470972
trans-4-hydroxy-L-proline 2	620	13.882,0	30	304	0.002175399	0.004165289	1.478255634	0.001741467	0.004794083	0.522268428	-0.937136602
Ethanolamine	913	10.4532,0	30	174	0.294677592	0.516125634	1.561688425	0.000780765	0.002499383	0.570941594	-0.808584926
D-galacturonic acid 2	663	18.651,0	30	292	0.031803935	0.054807424	1.321180855	0.020401391	0.031149414	0.580285158	-0.785166066
guanine 1	251	20.2692,0	27	267	0.016867281	0.028995265	1.546393446	0.002065427	0.005473892	0.581725367	-0.781589878
Phosphoglycolic acid	582	14.4788,0	29	299	0.10269136	0.157969841	1.16307669	0.035999455	0.047666389	0.650069402	-0.621334344
Myristic Acid	779	17.5477,0	30	117	0.010169677	0.01408986	1.370694744	0.016512323	0.026665025	0.721772763	-0.470383393
myo-inositol	853	19.9332,0	30	317	0.143364806	0.195139066	1.494649068	0.003338975	0.00788545	0.734680194	-0.444811713
pantothenic acid	873	18.9455,0	30	291	0.012610215	0.016808469	1.357240448	0.005474757	0.011924594	0.750229829	-0.41459547
glutamic acid	925	14.9919,0	30	246	0.53020842	0.70445575	1.47628077	0.002783422	0.00685173	0.752649717	-0.409949505
creatine	620	14.2274,0	29	220	0.462021688	0.596208394	1.475636981	0.002502	0.006313654	0.774933215	-0.367856114
6-phosphogluconic acid	666	22.6914,0	30	318	0.15133722	0.19444415	1.171075132	0.026659964	0.038349118	0.778306885	-0.361588975
Purine riboside	709	22.5942,0	30	217	0.018303265	0.023017487	1.087189017	0.044977591	0.0562077	0.795189526	-0.330629341
oxoproline	824	13.8995,0	30	258	0.867389838	1.08570054	1.582051922	0.000818339	0.002593705	0.798921807	-0.323873785
Fructose 2,6-biphosphate degr prod 2	671	20.9922,0	30	227	0.353119713	0.431680869	1.366607431	0.008056121	0.016087169	0.818011032	-0.289807795
aspartic acid 1	919	13.8268,0	30	232	1.047546561	1.231073106	1.291358962	0.013387327	0.023164409	0.85092149	-0.232902067
stearic acid	926	21.3162,0	30	117	0.633788843	0.725719266	1.126546532	0.043307937	0.054610928	0.87332509	-0.195409307
2-hydroxypyridine	895	7.17394,0	30	152	0.50509408	0.424943961	1.319763992	0.00830203	0.016447858	1.188613385	0.249279533
serine 1	933	11.7403,0	30	73	4.01992316	3.345204922	1.296543211	0.015414498	0.025489757	1.201697132	0.265073334
Erythrose 1	478	12.9376,0	30	350	0.028951562	0.023775039	1.453549488	0.003168518	0.007574922	1.217729313	0.284193475
ribose	893	15.6039,0	30	103	0.283370756	0.225828662	1.523754916	0.00095356	0.00291822	1.254804212	0.327462277

Bis(2-hydroxypropyl)amine 1	176	11.2165,0	29	341	0.002622943	0.002086571	1.246007663	0.017614682	0.027790978	1.257058594	0.330051898
2-deoxy-D-glucose 2	446	16.7263,0	30	319	0.00425983	0.003374116	1.129253675	0.031331097	0.043202	1.262502632	0.336286396
oxalic acid	691	8.49528,0	30	147	0.009006841	0.007099298	1.213848002	0.042051991	0.053390629	1.268694591	0.343344815
mannose 2	883	18.1856,0	30	160	0.978126085	0.766338072	1.436311108	0.002876765	0.007025319	1.276363685	0.352039467
proline	931	10.915,0	30	142	2.032631091	1.540911773	1.603356499	0.00042606	0.001522555	1.319109327	0.399564139
Diglycerol 1	370	16.4201,0	24	205	0.009439249	0.00706438	1.51174845	0.002239437	0.005818523	1.336175017	0.418108989
sarcosine	902	8.60011,0	30	116	0.030109051	0.022483839	1.196165537	0.027031464	0.038750367	1.339141896	0.421308837
2,3-Dimethylsuccinic acid	524	11.9438,0	30	247	0.002253233	0.001625659	1.251329105	0.017518975	0.027695279	1.386042424	0.470971416
4-Acetamidobutyric acid 1	265	13.4056,0	30	128	0.551764158	0.391152765	1.490543258	0.002036313	0.005414879	1.4106104	0.496319582
N-Acetyl-beta-D-mannosamine 1	820	19.7701,0	30	202	0.047414901	0.033089835	1.67835438	6.30859E-05	0.000341474	1.432914387	0.518952414
phenylalanine 1	927	15.0942,0	30	218	0.708990246	0.492659869	1.472770217	0.002425137	0.006171683	1.43910696	0.525173823
oxalacetic acid	226	12.8378,0	29	327	0.001307862	0.000895134	1.372733281	0.008105102	0.016159468	1.461080109	0.547035281
alanine 1	898	8.11267,0	30	116	4.441732279	3.033142268	1.725068038	1.91477E-05	0.000159631	1.464399585	0.55030927
6-Hydroxynicotinic acid	403	14.3477,0	30	268	0.028753232	0.019273505	1.139049981	0.048263881	0.059268329	1.491852751	0.577105145
methionine 1	693	13.7994,0	30	176	0.319677365	0.212993449	1.375124791	0.007738333	0.015612494	1.500878864	0.585807542
valine	944	9.73336,0	30	144	1.777477746	1.177132789	1.708240113	3.54992E-05	0.000237361	1.510006146	0.594554422
Isoleucine	940	10.8386,0	30	158	1.0091127	0.653741719	1.660262104	0.000109874	0.000520863	1.543595384	0.626294635
glycolic acid	889	7.70828,0	30	147	0.023271429	0.014997593	1.674009235	0.000195293	0.000805186	1.551677562	0.633828797
threonine 1	946	12.0667,0	30	219	0.856959538	0.548452523	1.68530469	0.000312428	0.001176759	1.562504505	0.643860349
Maleamate 1	406	14.333,0	30	244	0.004624039	0.002877379	1.468115013	0.003236119	0.007698983	1.607031601	0.684398299
3-Hydroxypropionic acid 1	422	8.7495,0	30	234	0.006950911	0.004325119	1.703624594	5.29882E-05	0.000308312	1.607102914	0.684462318
Glucoheptonic acid 1	351	21.6275,0	29	186	0.013254063	0.008139793	1.251826034	0.022770517	0.033976489	1.628304564	0.703370571
Threonic acid	772	14.035,0	30	292	0.006571077	0.003756775	1.564146469	0.000907199	0.002809506	1.749127185	0.806635197
citrulline 1	772	17.2579,0	30	256	0.017478599	0.009597667	1.477256942	0.002466475	0.006248335	1.821129864	0.864833804
beta-Mannosylglycerate 2	727	17.2939,0	30	217	0.070173238	0.037796711	1.486628994	0.002211112	0.00576335	1.856596434	0.892660252
D-Talose 1	927	17.8572,0	30	319	0.224875566	0.118003419	1.769335632	0.000433839	0.00154515	1.90566993	0.93029826
2-Deoxy-D-galactose 2	366	16.7491,0	29	292	0.00265293	0.001339563	1.563667973	0.001667567	0.004631565	1.980444865	0.985824538
Threitol	845	13.6103,0	30	217	0.037419175	0.017655238	1.72107446	4.24257E-05	0.000267851	2.119437633	1.083681514
shikimic acid	271	17.0767,0	18	231	0.001555609	0.000722667	1.12841607	0.049444561	0.06034217	2.152593668	1.106076016
2-hydroxybutanoic acid	912	8.42903,0	30	131	0.047232573	0.020562052	1.839533736	1.0102E-07	3.87893E-06	2.297074835	1.199797858
2,3-dihydroxybenzoic acid	274	16.3921,0	30	254	0.001216254	0.000519653	1.650720135	0.000103028	0.000495186	2.340512359	1.226824383
glutamine 2	292	16.0949,0	30	216	0.008807071	0.00370031	1.596015112	0.000579976	0.001961525	2.380090151	1.251016219
mannitol	875	18.3763,0	29	319	0.115082046	0.047506989	1.458703721	9.65952E-05	0.000470404	2.422423458	1.276451081
taurine	899	15.5638,0	30	326	0.538725308	0.206806618	1.620447562	0.004660247	0.010449731	2.604971316	1.381267486

2-Methylglutaric Acid	215	12.4972,0	28	172	0.005389937	0.001990387	1.873864512	2.4143E-08	1.79024E-06	2.707984018		1.437219225
3-Methyloxindole 2	296	14.1582,0	23	219	0.002285949	0.000724092	1.16108133	0.007018444	0.014499998	3.156988394		1.658548957
Guanidinosuccinic acid 4	261	19.9537,0	23	398	0.087967426	0.027293238	1.679596924	0.000121496	0.000563997	3.223048383		1.688425846
glucose-6-phosphate 1	819	21.8786,0	30	387	0.005930177	0.001696041	1.640092977	0.037427245	0.048975955	3.49648199		1.805904074
thymine	718	12.27,0	30	255	0.005358506	0.001450383	1.837191072	0.00013137	0.000599264	3.694545136		1.885396754
2,4-diaminobutyric acid 4	333	14.9653,0	29	331	0.068840226	0.016719071	1.856543698	1.8604E-09	4.49782E-07	4.117467175		2.041757149
MALONAMIDE 5	496	12.6889,0	24	89	0.053106277	0.012556203	1.85972358	2.54556E-08	1.8135E-06	4.229485424		2.08048215
uric acid	820	19.9851,0	30	442	1.144751718	0.268330606	1.797553804	1.2626E-06	2.02667E-05	4.266198838		2.092951208
5,6-dihydrouracil 1	422	13.3284,0	25	100	0.099253817	0.022290133	1.854379223	0.000219184	0.000872335	4.452813988		2.154717346
Glutaric Acid	545	12.3446,0	22	261	0.000817063	0.000176764	1.37168867	0.000335415	0.001247573	4.622336232		2.208622206
1,3-diaminopropane	332	16.5282,0	30	202	0.080540387	0.01515143	1.821555409	2.25585E-07	6.05988E-06	5.315695506		2.410258466
N-Acetyl-L-leucine 1	182	13.7389,0	23	197	0.001523009	0.000188585	1.624189156	0.000171094	0.000732065	8.075984772		3.01363819
Digitoxose 1	246	14.9958,0	17	69	0.009647704	0.001144052	1.410211736	1.66027E-05	0.000147135	8.432926781		3.076033428
Fluorene	308	15.1693,0	23	258	0.002370293	0.000204833	1.323624161	2.17448E-07	5.95822E-06	11.57183003		3.532545133
Adipamide 5	371	16.9787,0	25	204	0.003577104	0.000239861	1.480738123	1.54744E-05	0.000141077	14.91325075		3.898522862
2,5-dihydroxybenzaldehyde	179	15.8532,0	24	330	0.002955961	0.000182474	1.660791159	5.82327E-06	7.03937E-05	16.19931838		4.017861205
Atrazine-2-hydroxy 6	287	18.5926,0	16	357	0.02775076	0.001631184	1.635414655	4.66831E-05	0.00028496	17.01264797		4.088535805
N-alpha-Acetyl-L-ornithine 3	290	15.8142,0	29	188	0.020155049	0.001099357	1.183242877	3.8004E-05	0.000248795	18.33349343		4.196409811
succinate semialdehyde 1	706	9.30685,0	23	113	0.005502796	0.000289358	1.666572699	6.13154E-07	1.26431E-05	19.01726907		4.249238181
noradrenaline	395	20.594,0	22	128	0.002843847	9.25649E-05	1.344325724	0.01103395	0.020178894	30.72272626		4.941234338
N-formyl-L-methionine 1	262	16.0023,0	22	272	0.00141416	2.67276E-08	1.894035371	5.06013E-05	0.000299733	52910.08386		15.69125508
alpha-ketoisocaproic acid 1	511	9.69452,0	7	200	0.001547437	2.67276E-08	1.371673354	0.029817188	0.041673807	57896.57383		15.82119036
4-hydroxyphenylacetic acid	521	15.2488,0	12	179	0.001753596	2.67276E-08	1.892431425	0.00079226	0.002528439	65609.91768		16.00162629
alpha-ketoglutaric acid	533	14.4595,0	11	198	0.002217391	2.67276E-08	1.347725747	0.038250964	0.049719456	82962.58431		16.34017321
nornicotine	390	14.117,0	17	142	0.002959982	2.67276E-08	1.337111472	0.029911858	0.041770589	110746.2489		16.75689831
3-Phenyllactic acid	602	14.5772,0	18	193	0.006018325	2.67276E-08	1.890879762	0.004377449	0.009917111	225172.6306		17.78067195
canavanine 1	323	19.0176,0	14	204	0.014224865	2.67276E-08	1.893695028	0.000506171	0.001748221	532216.2134		19.02165294
