# Supplementary material for: A Multi-Omics Study of Chicken Infected by Nephropathogenic Infectious Bronchitis Virus
Source: Viruses. 2019 Nov 16;11(11):1070. doi: 10.3390/v11111070 (PMC6893681; doi:10.3390/v11111070)
Supplement: Supplementary file 1 [file viruses-11-01070-s001.zip › Supplementary files-for conversion/Table S3.pdf]

Supplementary Table S3. Basic summary of sequence and sequencing reads mapping to reference genome.

Sample name	Nor1	Nor2	Nor3	Nor4	Dis1	Dis2	Dis3	Dis4
Clean reads	70634494	62725636	72022668	65347746	63184400	69714812	74909822	65748400
Clean bases	10540771138	9354925902	10738879816	9747412458	9420602814	10384745730	11178689528	9809949868
GC content（%）	52.14%	52.00%	52.34%	51.70%	52.35%	52.99%	53.09%	52.76%
≥Q30%	90.59%	90.50%	90.70%	90.53%	90.75%	90.37%	90.48%	90.46%
Mapped reads	55703112	49556909	57103776	51486983	48881880	54505329	56625975	51372990
Uniq mapped reads	48693955	42815884	49482548	47049534	44148391	47217471	51416673	44615714
Multiple map reads	7009157	6741025	7621228	4437449	4733489	7287858	5209302	6757276
Reads map to'+'	27362658	24319365	28014121	25374162	23987517	26671432	25857823	25161253
Reads map to'-'	27091663	23971886	27734642	25279359	23986878	26462043	27714491	24957413
