# Supplementary material for: A Multi-Omics Study of Chicken Infected by Nephropathogenic Infectious Bronchitis Virus
Source: Viruses. 2019 Nov 16;11(11):1070. doi: 10.3390/v11111070 (PMC6893681; doi:10.3390/v11111070)
Supplement: Supplementary file 1 [file viruses-11-01070-s001.zip › Supplementary files-for conversion/Table S4.pdf]

Supplementary Table S4. DEGs in enriched pathways for correlation analysis.

Pathway	Gene name	Con1	Con2	Con3	Con4	Dis1	Dis2	Dis3	Dis4
Alanine, aspartate and glutamate metabolism	NIT2	71.603132	80.865232	74.095905	89.600993	32.3776	17.64349	30.501206	33.4303426
	ABAT	312.40373	303.81609	333.7616305	326.313048	36.94086524	46.72609726	47.02183771	76.58192001
	ALDH4A1	769.846	802.751	1064.741	912.107	327.064	179.2577	210.536	405.55
	GLUL	2.832552	2.498347516	1.77183847	2.159829	15.450736	5.904455	14.152556	5.002988
	GPT2	93.7561	94.2833	93.5824	73.2361	691.692	645.451	714.117	836.649
	GFPT2	2.304530714	1.491460928	1.593819808	2.420851819	5.148460257	4.822846442	7.228362908	3.502842035
	IL4I1	0.440804212	0.030207	0.0536097	0	65.2104	10.38933	10.69482	23.59575
	ASNS	8.765867783	7.802552	6.047259814	7.546842938	56.047583	26.926292	41.8027469	29.2936741
	ADSS	13.55348983	12.03451	13.2820522	12.39195173	47.05024518	30.20439789	30.31583663	34.12430689
Valine, leucine and isoleucine biosynthesis	BCAT1	65.0789	73.1019	64.0636	78.6691	19.7189	29.6001	22.7196	30.9857
	SDSL	247.464	297.526	352.986	319.334	89.4963	84.7367	68.2857	124.5895
Arginine and proline metabolism	CKB	289.5898	244.5941	324.1446	405.3524	122.0761	103.2929	90.25225	222.2465
	C10ORF65	505.988	553.747	841.974	636.92	168.385	58.8952	148.66	204.672
	ALDH9A1	380.3040054	390.1440071	382.9430064	510.3390062	124.4530053	89.8085047	99.95950339	159.7490055
	GATM	572.37404	595.4976	655.2527044	488.2999513	11.82656548	18.52691922	13.06015173	16.5508277
	RP11-162P23.2	423.3867	528.1900116	514.875219	533.4394	203.0698	140.619477	207.2379	192.7922
	NOS1	4.395263335	6.0244273	6.4434526	4.8750543	0.305226761	0.62194768	0.507257873	2.5615011
	MAOB	32.5755	25.0599	28.9006	31.7521	9.46501	8.8452	10.4174	8.6347
	MAOA	16.04171313	14.35917145	26.10310012	17.25202598	5.08764691	8.68289954	6.326099851	7.648744
	AGMAT	56.758638	73.75407	89.87713	51.87863	15.884174	12.31735	13.414976	25.630151
	GAMT	544.669287	581.88673	622.4979	715.911	5.93929	44.843917	16.74010007	31.17090027
	ALDH7A1	170.789	179.939	203.416	209.251	66.6884	51.8529	63.8835	74.4329
	PRODH2	415.201	685.11	569.377	520.441	38.2354	43.9815	56.1652	72.4157
	CNDP1	7.379936504	16.91711926	18.95411533	17.51504971	1.935718974	4.94030656	3.292934317	3.243590986
	CKMT1A	0.2011842	0.40097013	0.1871124	0.325215	2.925215	1.686977	1.907165	3.177458
	AMD1	17.2087	14.6945	15.4666	18.8804	49.4648	34.5065	43.8706	43.9003
	SMOX	2.912060694	3.203080909	3.754452062	3.625748852	9.840825227	10.4087025	7.812892357	7.349482632
Glutathione metabolism	SRM	12.360831	12.89892	10.29326	10.14283	31.885431	29.541421	30.454784	22.985432
	RRM1	8.053977	5.728037	6.578884052	4.866717	15.562669	18.75138968	15.507774	11.90605952
	GPX2	5.30169	2.56245	2.70851	3.47838	27.7109	44.1503	19.6374	18.3542
	ODC1	14.18965687	11.6022817	12.72195	11.725163	67.77228	52.03775	54.27774	47.430328
	GSTO1	16.42423964	22.26502	17.13582	21.7016223	43.76257	43.72796707	39.56811327	37.6798
	RRM2	29.2875	24.74863	37.4647	12.23911	44.781	89.451	55.56502	56.3835
	GSS	104.2556	105.9655	151.0077	121.8454	45.53765	46.3694	44.74865	73.45052
	GSTA3	535.2008	300.0757	422.911	505.351	109.1063	72.4012	31.51924	69.4909
	SMS	91.709307	93.83137629	86.44696822	93.19073043	30.30695035	29.95071912	32.75486128	38.18907269
	GSTA4	410.1197553	332.949756	185.60031	243.313726	75.5452128	177.1804401	89.607263	79.72482041
	GCLC	59.19261	46.72996	48.14314	54.29354	18.10372	27.81567	18.87879	26.95386
	ANPEP	415.0968	363.3225	448.9513	482.00332	111.44613	163.5249	94.53399	153.5575

Taurine and	CDO1	378.465	294.257	389.387	332.751	50.6474	115.51	46.9607	92.1066
hypotaurine	BAAT	253.269	201.2751	235.896	212.0223	66.6602	105.1931	45.2679	82.8542
metabolism	GGT1	96.243928	98.030581	83.28924	95.634978	252.3137	200.47347	163.039183	181.912703
Toll-like receptor	MAP2K6	4.25629556	4.59053833	6.18478103	6.061521672	0.729185	1.591598573	1.499387928	1.553729816
signaling pathway	NFKBIA	36.9569	17.5547	26.0391	21.8563	92.7917	72.0545	78.2277	50.1689
	FOS	65.9663	14.1148	17.4933	14.0301	74.7772	110.918	70.7239	50.3106
	STAT1	62.439	14.82118	17.46202	14.93301	114.5458	54.9086	69.7714	126.9416
	CD86	0.530476791	0.338996751	0.127733322	0.262139446	1.675709094	1.441345	1.121094137	2.069842813
	JUN	63.1099	29.1786	38.3154	31.8193	199.457	165.682	118.67	137.015
	IRF1	65.85490771	14.64216029	13.752105	11.49495	148.8513094	63.39996471	106.8813211	141.1075395
	CD80	1.61927	0.289929	0.629646	0.43863	9.14804	7.02659	7.83021	4.72385
	TLR4	1.431777	0.669567	0.745925	0.470317697	10.26368	9.25241	4.41967	5.70615
	PIK3R5	3.8251775	1.196221544	1.823405495	1.317520716	17.8592662	8.6167511	11.9070785	10.08818238
	PIK3CD	2.780914	1.285505533	0.975937111	0.932581	7.547614893	6.21895825	6.444042	5.058966196
	TLR7	0.913652952	0.345442131	0.714439863	0.791350791	6.581399985	4.026234216	2.036750878	2.857612
	IRF5	1.58835986	1.26338298	0.8661456	1.089538918	12.8768361	11.1654243	5.969877635	7.387919271
	MYD88	23.84632606	19.998613	16.55749307	15.37328267	40.764662	38.68185101	33.187205	40.33444185
	MAP3K8	0.368374857	0.093703747	0.171752861	0.062301188	2.465799022	2.229510206	2.192733722	1.90529169
	IRF4	2.856606759	1.014937914	1.112596907	0.275859062	7.608422773	11.18227539	25.72109291	5.628806094
	LY96	4.028964389	1.257109275	1.183611399	0.774908492	33.33173406	13.34970155	17.56247683	18.83713398
	TLR2B	1.37174812	0.879439	0.6362068	0.8641868	5.578446	6.210908	2.963125	6.811834726
	SPP1	633.184	714.714	904.752	632.161	32879.3	14296.3	19221.8	23300.6
	TLR1B	2.606954	1.08469	1.992327	1.132797	27.3045	13.72107	7.86745	11.15004
	IL12B	0.104763513	0.41572533	0.094278016	0.091346082	12.38135769	1.147767994	8.7655963	6.070706109
	TLR1A	0.84342565	0.426374178	0.398132805	0.152420965	2.76800307	1.18052614	3.183935541	4.140815231
RIG-I-like receptor	DAK	133.054	126.5485	145.6295	133.2798	31.5293	62.8993	42.512	39.4742
signaling pathway	TRAF3	1.760503664	1.044786563	1.31290343	1.262775537	6.428068734	4.163610317	5.728049348	4.037333738
	CASP8	2.51719918	1.8250898	1.742102991	2.366821309	6.786018953	5.221523855	4.027567279	5.402663192
	IFIH1	10.1824	4.11908	3.65955	3.51363	25.817	12.9088	14.8276	38.0059
	TRAF2	11.39488676	8.540602896	9.57905075	8.317487329	23.9925383	21.50245915	22.21927644	22.30211461
	TRIM25	16.52735728	5.839458	9.044854581	6.568616782	29.16190226	19.88690824	23.08043673	41.05184008
	IKBKE	2.18057219	1.152881155	1.89486	1.096546411	9.044769337	5.532039436	5.654477794	7.193297487
	MAPK11	8.752827325	10.00947	7.644642	9.694101	29.52769	16.513337	18.95018	17.0193
	RIPK1	6.606422023	5.121945493	4.642057896	5.599313003	14.5102531	10.88786411	11.480688	11.54852283
	IL8L1	0.234313	0.134523	0.316399	0	7.07631	3.15695	4.82439	2.7661
	IL8L2	0.127245	0	0.128063	0.0905099	8.2915	2.69807	6.03598	3.75036
	IRF7	40.7185	11.0478	17.4223	10.6913	73.1037	37.8967	50.6377	64.2461
NOD-like receptor	PSTPIP1	1.825958	0.746105	0.713967	0.674481	6.42951	4.772357	3.829956	4.870013
signaling pathway	RIPK3	0.606444001	0.918943409	0.762654504	0.52931981	4.5813074	3.100644185	2.655739501	2.9135843
	BIRC2	23.18757916	16.39102687	14.99002373	17.61406048	76.7819778	42.7203593	57.761172	57.4761153
	NOD1	1.791660149	2.253268	1.8076949	1.941178	4.486049	5.253927	3.4185654	5.267261
	HSP90AB1	9.07988878	6.716221	5.400646533	7.038127913	15.67071128	15.22992492	21.01133802	9.256559302
	TNFAIP3	3.26323482	1.2780938	1.955257679	1.769867823	10.20529042	7.76736601	8.363994236	7.340162
	IL18	0.127245	0	0.128063	0.0905099	8.2915	2.69807	6.03598	3.75036
