# Supplementary material for: A Multi-Omics Study of Chicken Infected by Nephropathogenic Infectious Bronchitis Virus
Source: Viruses. 2019 Nov 16;11(11):1070. doi: 10.3390/v11111070 (PMC6893681; doi:10.3390/v11111070)
Supplement: Supplementary file 1 [file viruses-11-01070-s001.zip › Supplementary files-for conversion/Table S5.pdf]

Supplementary Table S5. Differentially abundant metabolites in enriched pathways for correlation analysis.

Pathway	Peak	Con1	Con2	Con3	Con4	Con5	Con6	Dis1	Dis2	Dis3	Dis4	Dis5	Dis6
Alanine, aspartate and glutamate metabolism	oxalacetic acid	0.00069376	0.000775381	0.00111535	0.00101158	0.000985463	0.00078927	0.001663509	0.000925736	0.001222204	0.001200864	0.00151636	0.001318499
	aspartic acid	1.245602617	1.115647598	1.169873277	1.344638615	1.315988664	1.194687867	0.997435413	1.207555147	0.894560244	1.174310246	1.041881507	0.969536812
	glutamine	0.006504834	0.004754972	0.002107914	0.002702488	0.003454423	0.002677228	0.010017281	0.009867015	0.011405403	0.007461883	0.006345433	0.007745412
Valine, leucine and isoleucine biosynthesis	alpha-ketoisocaproic acid	2.32584E-08	2.43822E-08	2.46245E-08	2.68561E-08	2.81891E-08	3.30552E-08	0.001808934	0.002014879	2.86198E-08	0.002908737	2.75615E-08	0.002552014
	valine	1.347067655	0.95798684	1.100705123	1.328439253	1.319294501	1.009303362	1.969645684	1.828905948	1.73089642	1.703842288	1.646863532	1.784712606
	Isoleucine	0.756467046	0.51328024	0.615290369	0.7644325	0.745037067	0.527943092	1.163672517	0.997390744	0.950799299	1.004283569	0.939901522	0.998628549
	threonine	0.639825259	0.477412263	0.511098378	0.57224298	0.607332733	0.482803525	0.895383097	1.064144738	0.726852101	0.871724753	0.73855603	0.845096511
Arginine and proline metabolism	sarcosine	0.026200365	0.021030481	0.015067966	0.020069122	0.026695531	0.025839572	0.027126155	0.031256197	0.039481909	0.032399634	0.023963886	0.026426527
	proline	1.809536368	1.380092931	1.414305467	1.614864375	1.732238802	1.294432694	2.129820516	2.078096425	1.869587314	1.946557224	2.13341911	2.038305959
	4-Acetamidobutyric acid	0.443652301	0.368966921	0.322735892	0.444951852	0.468452029	0.298157594	0.600173983	0.522831342	0.445218888	0.608786123	0.595380281	0.538194332
	trans-4-hydroxy-L-proline	0.003554071	0.003723472	0.003975702	0.003903037	0.005710748	0.004124704	0.001106948	0.001452241	0.002328569	0.002758136	0.003406049	0.002000451
	creatine	0.652634916	0.639044267	0.533070728	0.601087609	0.660223668	0.491189179	0.53004083	0.449339273	0.491131763	0.403062037	0.456401113	0.442155112
	1,3-diaminopropane	0.022866514	0.01161995	0.01995071	0.016753506	0.010162768	0.00955513	0.089788665	0.077093926	0.093719784	0.080220754	0.060162905	0.082256289
Taurine and hypotaurine metabolism	alpha-ketoglutaric acid	2.32584E-08	2.43822E-08	2.46245E-08	2.68561E-08	2.81891E-08	3.30552E-08	0.002276523	0.003163532	2.86198E-08	2.39197E-08	0.002864803	0.004999436
	taurine	0.264335014	0.151974736	0.161436719	0.166132743	0.208823474	0.288137022	0.394439899	0.573998774	0.776059348	0.309253305	0.487448998	0.691151523
Glutathione metabolism	oxoproline	0.978397115	1.133065815	0.957530716	1.163982759	1.134511874	1.14671496	0.839841805	0.935837837	0.814526421	0.964857603	0.810364122	0.838911237
	glutathione	0.049523975	0.071254053	0.027050411	0.048412323	0.034008427	0.012808341	0.006364589	0.008683468	0.003772334	0.009364897	0.008492777	0.006441679
others	phenylalanine	0.643322328	0.399856889	0.426208142	0.555333741	0.572795858	0.358442253	0.772510391	0.72916533	0.613069789	0.789921659	0.668087546	0.681186762
	beta-Mannosylglycerate	0.04469574	0.054241862	0.023946093	0.036955535	0.037968563	0.02897247	0.068155819	0.073446344	0.040582493	0.078567108	0.071773785	0.088513879
	uric acid	0.318763088	0.16704227	0.192946018	0.288602988	0.434143096	0.208486175	1.188426024	1.27935287	1.237294221	0.783340446	1.135820205	1.244276545
	guanine	0.02759396	0.036703792	0.022421724	0.026228451	0.034371404	0.026652261	0.014538958	0.026263732	0.016747178	0.015715016	0.014682808	0.013255997
	2-hydroxybutanoic acid	0.019864606	0.018336624	0.017340744	0.020016743	0.022931839	0.024881757	0.050434261	0.049984603	0.04938632	0.048128579	0.040065003	0.045396672
	thymine	0.001308585	0.001536876	0.001792925	0.001300083	0.001706634	0.001057195	0.005353691	0.004166249	0.004930615	0.004783609	0.005803243	0.007113627
	2-Methylglutaric Acid	0.001831177	0.002012992	0.00178918	0.00207314	0.002310606	0.001925229	0.005943169	0.005153906	0.005031716	0.004946772	0.005161338	0.006102723
	malonamide	0.017655895	0.012234328	0.012825913	0.012497339	0.009715402	0.01040834	0.052679766	0.056004286	0.056889343	0.056900387	0.041812956	0.054350923
	5,6-dihydrouracil	0.027501838	0.017922347	0.022768039	0.022963891	0.022315751	0.020268934	0.111049907	0.098244591	0.107836882	0.068634962	0.083435387	0.126321175

N-Acetyl-L-leucine	2.32584E-08	2.43822E-08	2.46245E-08	2.68561E-08	0.001131378	3.30552E-08	0.001634826	0.001857144	0.001167163	0.001911199	0.001259605	0.001308118
3-Phenyllactic acid	2.32584E-08	2.43822E-08	2.46245E-08	2.68561E-08	2.81891E-08	3.30552E-08	0.009178447	0.002247432	0.009872479	0.00399107	0.006074694	0.00474583
2,4-diaminobutyric acid	0.022603107	0.015262196	0.011330833	0.016540789	0.018036951	0.01654055	0.066925656	0.07017107	0.067588549	0.072944019	0.074913143	0.060498922
4-hydroxyphenylacetic acid	2.32584E-08	2.43822E-08	2.46245E-08	2.68561E-08	2.81891E-08	3.30552E-08	0.00163529	0.00269735	0.001217355	0.001694655	0.002160488	0.001116437
N-formyl-L-methionine	2.32584E-08	2.43822E-08	2.46245E-08	2.68561E-08	2.81891E-08	3.30552E-08	0.001592506	0.001630243	0.001072844	0.001680693	0.001405279	0.001103393
3,6-Anhydro-D-galactose	0.096815713	0.092099465	0.065536342	0.084598038	0.092098967	0.071028205	0.007144997	0.005744668	0.00837207	0.006746821	0.010048667	0.007332523
canavanine	2.32584E-08	2.43822E-08	2.46245E-08	2.68561E-08	2.81891E-08	3.30552E-08	0.020799683	0.01303014	0.009838741	0.015776946	0.016584108	0.009319571
noradrenaline	0.000555252	2.43822E-08	2.46245E-08	2.68561E-08	2.81891E-08	3.30552E-08	2.72188E-08	0.005267914	0.002794963	0.00336318	0.003401643	0.002235354
