# Supplementary material for: A Multi-Omics Study of Chicken Infected by Nephropathogenic Infectious Bronchitis Virus
Source: Viruses. 2019 Nov 16;11(11):1070. doi: 10.3390/v11111070 (PMC6893681; doi:10.3390/v11111070)
Supplement: Supplementary file 1 [file viruses-11-01070-s001.zip › Supplementary files-for conversion/Table S6.pdf]

Supplementary Table S6. The relative abundance of 7 discriminative features (LDA score ≥ 4) identified by LEfSe analyse.

Class	Con1	Con2	Con3	Con4	Con5	Con6	Dis1	Dis2	Dis3	Dis4	Dis5	Dis6
Bacteria|Bacteroidetes|Bacteroidia|Bacteroidales|Bacteroidaceae	0.440737705	0.218893443	0.123442623	0.298504098	0.177971311	0.124590164	0.015901639	0.198401639	0.095942623	0.022418033	0.033913934	0.156536885
Bacteria|Bacteroidetes|Bacteroidia|Bacteroidales|Bacteroidaceae|Bacteroides	0.440737705	0.218893443	0.123442623	0.298504098	0.177971311	0.124590164	0.015901639	0.198401639	0.095942623	0.022418033	0.033913934	0.156536885
Bacteria|Bacteroidetes|Bacteroidia|Bacteroidales|Bacteroidaceae|Bacteroides|Bacteroides_vulgatus	0.369815574	0.198135246	0.106454918	0.253668033	0.154713115	0.086577869	0.011885246	0.094303279	0.06692623	0.007377049	0.007704918	0.14432377
Bacteria|Firmicutes|Bacilli|Lactobacillales	0.068934426	0.087356557	0.017233607	0.033463115	0.045102459	0.067479508	0.046803279	0.04352459	0.00375	0.004795082	0.011290984	0.008852459
Bacteria|Firmicutes|Bacilli|Lactobacillales|Lactobacillaceae	0.05954918	0.084364754	0.015102459	0.022377049	0.038934426	0.062704918	0.033565574	0.031270492	0.0025	0.00375	0.011106557	0.007827869
Bacteria|Firmicutes|Bacilli|Lactobacillales|Lactobacillaceae|Lactobacillus	0.05954918	0.084364754	0.015102459	0.022377049	0.038934426	0.062704918	0.033565574	0.031270492	0.0025	0.00375	0.011106557	0.007827869
Bacteria|Proteobacteria	0.018258197	0.005286885	0.010348361	0.010737705	0.023319672	0.024159836	0.614795082	0.048852459	0.039836066	0.111372951	0.057848361	0.008278689
